# Intervention to Improve Quality of life for African-AmericaN lupus patients (IQAN): study protocol for a randomized controlled trial of a unique a la carte intervention approach to self-management of lupus in African Americans

**DOI:** 10.1186/s12913-016-1580-6

**Published:** 2016-08-02

**Authors:** Edith M. Williams, Kate Lorig, Saundra Glover, Diane Kamen, Sudie Back, Anwar Merchant, Jiajia Zhang, James C. Oates

**Affiliations:** 1 Department of Public Health Sciences, Department of Medicine, Division of Rheumatology, Core Investigator, MUSC Center for Health Disparities Research, Medical University of South Carolina, 135 Cannon Street, Suite 303, MSC835, Charleston, SC 29425 USA; 2 Department of Medicine, Stanford University, 291 Campus Drive, Room LK3C02, Stanford, CA 94305 USA; 3 Institute of Health Disparities, University of South Carolina, 220 Stoneridge Drive, Suite 103, Columbia, SC 29210 USA; 4 Department of Rheumatology and Immunology, Medical University of South Carolina, 96 Jonathan Lucas Street, Suite 912, Charleston, SC 29425 USA; 5 Department of Psychiatry/Behavioral Science, Division of Clinical Neuroscience, Medical University of South Carolina, 67 President Street, Charleston, SC 29425 USA; 6 Department of Epidemiology and Biostatistics, University of South Carolina, 800 Sumter Street, Room 211, Columbia, SC 29208 USA; 7 Department of Epidemiology and Biostatistics, University of South Carolina, 915 Greene Street, Columbia, SC 29208 USA; 8 Department of Medicine, Division of Rheumatology, Medical University of South Carolina, 114 Doughty Street, Room 425-C, Charleston, SC 29414 USA

**Keywords:** Lupus, Quality of life, Self-management, African-American, Randomized trial

## Abstract

**Background:**

Systemic Lupus Erythematosus (lupus) is a chronic autoimmune disease that can impact any organ system and result in life-threatening complications. African-Americans are at increased risk for morbidity and mortality from lupus. Self-management programs have demonstrated significant improvements in health distress, self-reported global health, and activity limitation among people with lupus. Despite benefits, arthritis self-management education has reached only a limited number of people. Self-selection of program could improve such trends. The aim of the current study is to test a novel intervention to improve quality of life, decrease indicators of depression, and reduce perceived and biological indicators of stress in African-American lupus patients in South Carolina.

**Methods/Design:**

In a three armed randomized, wait list controlled trial, we will evaluate the effectiveness of a patient-centered ‘a–la-carte’ approach that offers subjects a variety of modes of interaction from which they can choose as many or few as they wish, compared to a ‘set menu’ approach and usual care. This unique ‘a-la-carte’ self-management program will be offered to 50 African-American lupus patients participating in a longitudinal observational web-based SLE Database at the Medical University of South Carolina. Each individualized intervention plan will include 1–4 options, including a mail-delivered arthritis kit, addition and access to an online message board, participation in a support group, and enrollment in a local self-management program. A ‘set menu’ control group of 50 lupus patients will be offered a standardized chronic disease self-management program only, and a control group of 50 lupus patients will receive usual care. Outcomes will include changes in (a) health behaviors, (b) health status, (c) health care utilization, and (d) biological markers (urinary catecholamines).

**Discussion:**

Such a culturally sensitive educational intervention which includes self-selection of program components has the potential to improve disparate trends in quality of life, disease activity, depression, and stress among African-American lupus patients, as better outcomes have been documented when participants are able to choose/dictate the content and/or pace of the respective treatment/intervention program. Since there is currently no “gold standard” self-management program specifically for lupus, this project may have a considerable impact on future research and policy decisions.

**Trial registration:**

NCT01837875; April 18, 2013

## Background

### Lupus disease experience of African Americans

Systemic Lupus Erythematosus (SLE) is a chronic autoimmune disease with acute periodic flare-ups of symptoms impacting any organ system and resulting in potentially life-threatening complications [1, 2]. SLE disproportionately affects nonwhites [3], and a number of studies have shown that African-Americans are at increased risk for morbidity and mortality from SLE [4–9]. In these studies, SLE occurrence was three to four times higher among African-American than Caucasian white women, and high levels of disease activity are more commonly observed in African-Americans [8–10]. Other significant complications of treatment include weight gain, osteoporosis, osteonecrosis, accelerated atherosclerosis, and retinal damage [1, 2, 11, 12]. These side effects and complications can lead to significant functional and emotional challenges. Patients often experience a high degree of psychological symptoms, including anxiety, depression, mood disorders, and decreased health-related quality of life [13–18]. In addition to managing disease-specific stressors, it has been suggested that African-Americans are exposed to a unique set of risk factors that lead to a pattern of cumulative disadvantage over time. High rates of unemployment, poverty, violent crime, incarceration, and homicide among African-American adults reflect this accumulation of disadvantage at multiple transition points during their development and across the life course [19–31]. It is highly likely that early childhood exposure to segregated, economically impoverished neighborhoods created by institutionalized racism adversely affects child health and growth and sets the Black child on a low education and economic trajectory that increases the risk of poor physical and mental health in adulthood [22]. Additional stressors include deprivation of resources and facilities, differential exposure to health risks in the physical environment because of economically disadvantaged neighborhoods and poor quality housing, higher costs of goods and services in deprived areas, as well as roles of social networks and social capital, which often give rise to peer pressure against academic achievement and in support of crime and substance use [19, 20, 23, 24]. Due to the exposure of African-Americans to a unique trajectory of stressors throughout the life course, it may be critical to address modifiable risk factors for SLE that may be further exacerbated by this trend in an effort to improve health status and reduce health disparities in this high risk group.

### Evidence based prevention programs

A large body of evidence has shown that health-promoting programs in stress management have been successful in helping people improve their health practices and related health conditions [25]. Based on reviews of scientific literature, investigators have suggested that therapeutic interventions should be proposed to reduce psychological distress to improve quality of life and possibly moderate the evolution of chronic and unpredictable diseases like SLE [32]. Cognitive-behavioral stress management (CBSM) techniques have resulted in short-term improvement in pain, psychological function, and perceived physical function among persons with SLE [27]. Additionally, psychoeducation [33] and graded aerobic exercise [34] have been shown to be useful in the management of fatigue. Programs designed to reduce stress levels of chronically ill patients have also included support therapy, lifestyle interventions incorporating elements of yoga or other similar disciplines, and mini-sessions on depression, adaptive coping strategies, and body image [27–29]. Although there is no generally accepted self-management program available for SLE [16], two programs that have been shown to be successful in improving conditions in patients with arthritis are the Arthritis Self- Management Program (ASMP) and the generic Chronic Disease Self-Management Program (CDSMP). Each program incorporates six weeks of peer led sessions ranging in disease-specific and more general self-help content. Arthritis self-management education delivered by small-group, home study, computer, and Internet modalities have demonstrated significant improvements in health distress, self-reported global health, and activity limitation, with trends toward improvement in self efficacy and mental stress management [31, 35–57].

### Barriers to participation

Despite the apparent need for help with multiple illness-related problems and evidence that some of these problems can be ameliorated with cognitive-behavioral interventions without adverse effect, several studies have emphasized the need to design interventions that address barriers to participation and curtail noncompliance [58–61], particularly for African-American patients. Practicing physicians continue to struggle with patient compliance, poor adherence to therapeutic regimens, and failure of patients to keep scheduled appointments. For example, Petri et al. (1991) found that physicians rated African-Americans as less globally adherent than whites (43.5 % versus 66.3 % adherent, respectively) [62].

Despite recommendations from numerous national agencies that self-management education complement medical care [25, 43–47], arthritis self-management education has reached a limited number of people. Many Arthritis Foundation chapters have had difficulty disseminating arthritis self-management education programs. Additionally, many vulnerable populations have not been included in study samples [40, 42, 45, 51, 63–66]. Compliance is also a persistent problem in standardized programs. One study reported that less than 50 % of a closed eligible population participated, even when Internet and small-group programs were offered repeatedly over many years [67], suggesting that interventions may not be reaching the largest portion of lupus cases. Self-selection of program components has not been explored as an approach to improve such trends, but better self-management outcomes have been documented when participants are able to choose/dictate the content and/or pace of the respective treatment/intervention program [68–71].

### Study aims

The overall hypothesis of this proposal is that a culturally sensitive educational intervention which includes self-selection of program components will lead to improved quality of life and health status among African-American patients with lupus. Our specific aims are: 1) To provide an intervention that employs a patient-tailored approach and offers a variety of modes of interaction from which patients can choose; 2) Measuring efficacy, implementation, and adoption/maintenance, using the *Reach, Effectiveness, Adoption, Implementation, and Maintenance (RE-AIM)* framework, which offers a comprehensive approach to considering five dimensions important for evaluating the potential public health impact of an intervention [72–79]; and 3) To use previously collected data to characterize patient-centric barriers to care in African-American lupus patients.

Our approach maximizes chances of success in improving quality of life, decreasing indicators of disease activity and depression, and reducing perceived and biological indicators of stress among African-American lupus patients. In an effort to circumvent barriers to participation and approach this problem in a real-world fashion, we will incorporate the documented needs and desires of the target population into the design. In addition to filling a critical gap in steps to validate various hypothesized mechanisms in lupus, particularly in African Americans, who are at highest risk for the disease, such a preliminary investigation may demonstrate that this method effectively reduces perceived and biological indicators of stress and should be considered as an adjunct to standard medical care for this high risk group. Intervention program components will be largely adapted from the work and guidance of Dr. Kate Lorig, original creator of the internationally-adopted Arthritis Self-Management Program and a mentor on this proposal. Indicators of stress, depression, and quality of life will be evaluated among a cohort of African-American lupus patients participating in an ongoing SLE Clinic Database Project at the Medical University of South Carolina (MUSC). The availability of uniform data detailing the sociodemographic and disease profile characteristics of SLE Clinic Database participants and overarching RE-AIM framework will allow for the investigation of differences in those who choose not to participate, respond, or adopt program components.

## Methods/Design

### Study design

**Fig. 1:**
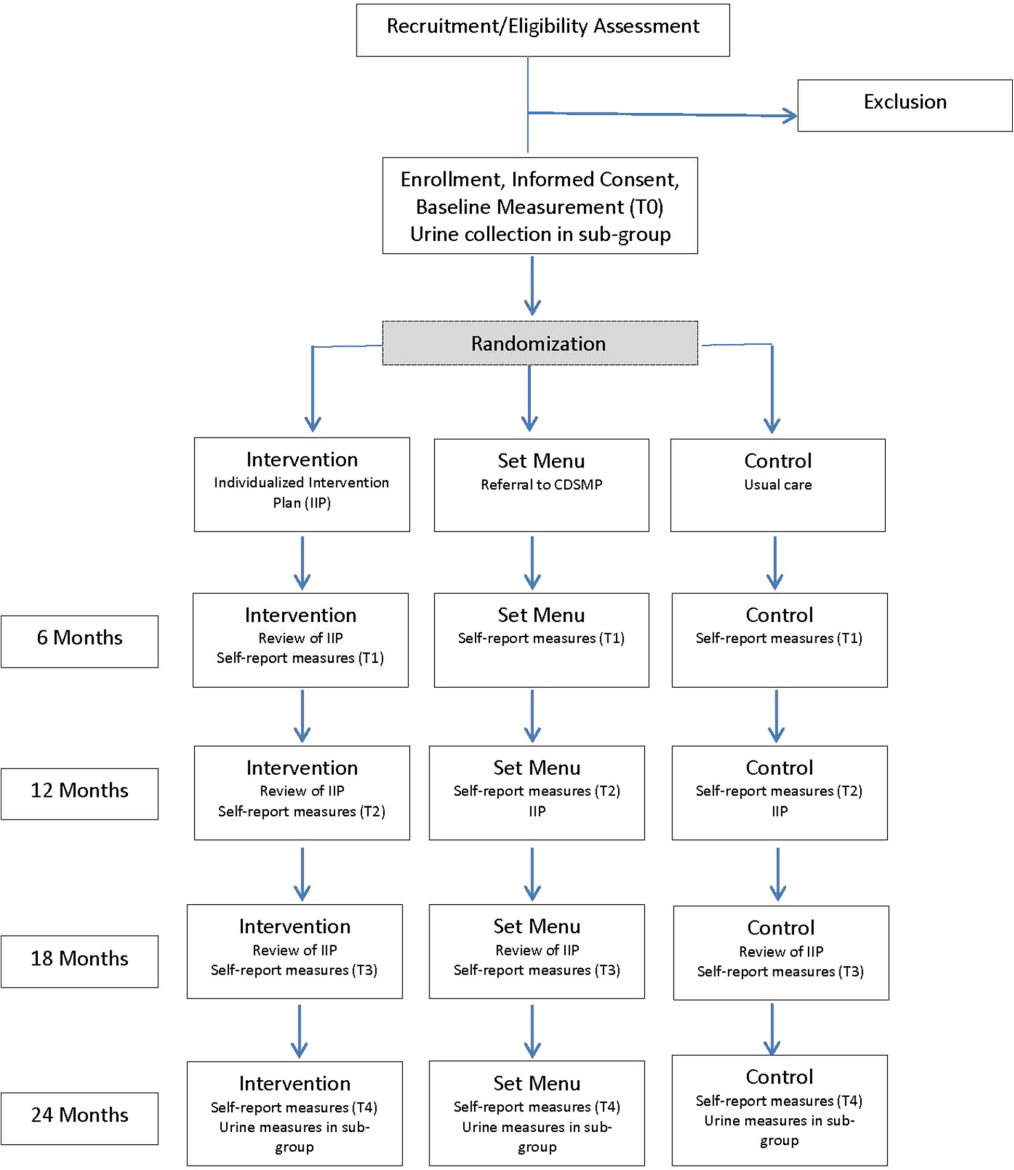
Study design

SPIRIT guidelines have been adhered to for the reporting of this protocol. As depicted in Fig. 1, IQAN is a three armed randomized, wait list controlled trial to compare a patient-centered ‘a–la-carte’ approach that offers subjects a variety of modes of interaction from which they can choose as many or few as they wish (IQAN intervention) to a ‘set menu’ approach. A unique ‘a-la-carte’ self-management program will be offered to 50 African-American lupus patients participating in an ongoing SLE Clinic Database Project at MUSC. A ‘set menu’ control group of 50 lupus patients will be offered a standardized chronic disease self-management program only, and a control group of 50 lupus patients will receive usual care (UC), void of intervention components. The intervention will be coordinated by an Intervention Coordinator, with assistance from local/MUSC Study Coordinators. The Intervention Coordinator will work with each participant in the intervention arm to create an individualized intervention plan (IIP). Each IIP will include 1–4 options, including a mail-delivered arthritis kit, addition and access to a listserv, participation in a support group, and enrollment in local self-management program(s). Due to the nature of the IIP, the proposed intervention will inherently vary by participant. To ensure intervention integrity, discrete components will follow written protocols and peer leaders will complete a standardized checklist for each session/encounter. The total number of study visits will depend on group assignment and intervention participation. The entire study is over a period of 2 years. Location will vary depending upon the randomly assigned intervention. Locations range from a home setting to a set community meeting location. We expect the proposed study to demonstrate that a unique intervention strategy can reduce disease activity and damage, as well as perceived and biological indicators of stress, while improving quality of life indicators in African-American lupus patients. Measures of psychosocial and neuroendocrine responses to stress and disease activity and damage indices are expected to be inversely related to participation in intervention activities. Indicators of quality of life are expected to be positively related to participation. Validated measures of stress, depression, and quality of life will be collected in all patients in each condition before and after intervention activities.

### Study setting and patients

The total number of individual patients with SLE currently being followed by clinicians at MUSC is 1,121 within the past 3 years. The total number of new patients with SLE seen in the past year by clinicians at MUSC was 176, of which 61 % were African-American and 88 % were female. Patients invited to participate in the proposed study are African-American lupus patients participating in a longitudinal observational web-based SLE Database at MUSC. There are 402 patients with lupus currently enrolled, and these patients are seen on a regular basis in the MUSC lupus clinics. All patients have American College of Rheumatology (ACR) criteria and disease activity information available, as well as quality of life measures obtained in the database questionnaire. All SLE patients met at least four components of the 1997 ACR revised criteria for SLE [80]. The vast majority of subjects have had serum/urine/DNA/RNA specimens collected, stored, and shared with collaborators across the U.S, resulting in many important discoveries in the genetics and epidemiology of lupus. The database is web-based, allowing quick identification of potential participants in clinical trials since, as part of the informed consent process, participants agree to future re-contact regarding other research studies. Of the 402 patients with lupus, 336 are African-American, and 218 of the 336 are Gullah African-American from the Sea Islands of South Carolina and Georgia. Additionally, as part of the associated SLE in Gullah Health (SLEIGH), 166 unrelated age- and gender-matched Gullah controls and 216 family-member Gullah controls are enrolled.

Target enrollment for the study is 150 patients who meet the following study eligibility criteria: a) have been diagnosed with lupus; b) be at least 18 years of age; c) free of central nervous system (CNS)/neuropsychiatric lupus and dementia; and d) not be a participant in the ASMP or CDSMP. Patients are excluded if they: a) are younger than 18 years of age; b) are not African American; c) do not have a confirmed diagnosis of lupus; d) have been diagnosed with CNS/neuropsychiatric lupus and dementia; and/or e) have been or are currently a participant in an ASMP or CDSMP. The study is registered at https://clinicaltrials.gov/ct2/show/NCT01837875?term=NCT01837875&rank=1: NCT01837875.

### Recruitment

Recruitment will be achieved on a systematic, randomized basis to be able to characterize respondents vs. non-respondents and more effectively document Reach or the percent and representativeness of individuals willing to participate. Three waves of recruitment will be implemented, each targeting up to 112 of the 336 African-American lupus patients currently enrolled in the MUSC SLE Database who have agreed to future contact. Recruitment efforts will be limited to participants residing in areas where self-management programs are offered who are free of CNS/neuropsychiatric lupus and dementia. Each wave will be one month in duration and include an initial mailed postcard describing the project and distribution of flyers with the same information during MUSC lupus clinic visits, both inviting interested patients to learn more about the proposed study, meet the investigative team, enroll in the study, and complete consent documents.

### Randomization

Within a month of all signed informed consent forms and baseline assessments being returned, the Biostatistician or their graduate assistant (GA) will enter baseline data and randomly assign each participant to one of three study arms: (1) intervention, (2) set menu control, or (3) control, returning that information to the principal investigator (PI) and Intervention Coordinator. Randomization will be stratified according to level of education (i.e., high school diploma or greater vs no diploma), or employment (more than 30 h per week vs less than 30 h), or income (SSI vs ≤ 25,000 vs > 25,000), depending on the completeness of data provided by the MUSC Study Coordinator. Within a month of randomization, the Intervention Coordinator will contact 50 intervention arm participants and assess and record their intervention modes of choice. The intervention coordinator will provide URL for listserv, support group details, and coordinate mailing of arthritis kits as applicable. The Intervention Coordinator will then link interested intervention arm and all set menu control arm participants to CDSMP’s in their area. The GA will make courtesy calls to the 50 participants in the control group letting them know of their designation and when they can expect contact and next round of evaluation materials to be completed and returned for compensation.

### Description of the intervention

The intervention will be coordinated by an Intervention Coordinator, with assistance from local/MUSC Study Coordinators. The Intervention Coordinator will work with each participant in the intervention arm to create an IIP. Each IIP will include 1–4 options, including a mail-delivered arthritis kit, addition and access to a listserv, participation in a support group, and enrollment in local self-management program(s). Due to the nature of the IIP, the proposed intervention will inherently vary by participant. The total number of study visits will depend on group assignment and intervention participation. The entire study is over a period of 2 years. Location will vary depending upon the randomly assigned intervention. Locations range from a home setting to a set community meeting location.

For the past 5 years, South Carolina has been moving toward statewide infrastructure development for evidence based prevention programs (EBPPs). Under the combined leadership of the South Carolina Department of Health and Environmental Control (SCDHEC) and the South Carolina Lieutenant Governor’s Office Administration (SCLGOA), a number of EBPPs have been integrated into the program delivery of many aging services organizations and other local partner organizations. The Intervention Coordinator will assist interested participants with enrollment, and participants will be encouraged to take advantage of other programs after study completion.

### Arthritis Foundation Self-Help Program

The Arthritis Foundation Self-Help Program (AFSHP) was the first patient education program developed by Stanford University. It is a 2-h workshop given weekly for six weeks in a variety of community settings, such as senior centers, churches, libraries, and hospitals. People with different types of rheumatic diseases, such as osteoarthritis, rheumatoid arthritis, fibromyalgia, lupus, and others, attend in a group format. Workshops are facilitated by two trained leaders, one or both of whom are non-health professionals with arthritis. Topics covered include: 1) techniques to deal with problems such as pain, fatigue, frustration and isolation, 2) appropriate exercise for maintaining and improving strength, flexibility, and endurance, 3) appropriate use of medications, 4) communicating effectively with family, friends, and health professionals, 5) healthy eating, 6) making informed treatment decisions, 7) disease related problem solving, and 8) getting a good night's sleep. It is the process in which the program is taught that makes it effective. Classes are highly interactive and engaging, where mutual support and success build the patients’ confidence in their ability to manage their health and maintain active and fulfilling lives.

### Chronic disease self-management – better choices better health

The Chronic Disease Self-Management Program (CDSMP) is a 2.5 h workshop given weekly for six weeks. Like the Arthritis Foundation Self-Help Program, it was developed by Stanford University and is also offered in a variety of community settings (e.g., senior centers, churches, hospitals). People with different chronic health problems attend together and support one another in making positive changes in their health. Workshops are facilitated by two trained leaders, one or both of whom are non-health professionals with a chronic disease themselves. Subjects covered include: 1) techniques to deal with problems such as frustration, fatigue, pain and isolation, 2) appropriate exercise for maintaining and improving strength, flexibility, and endurance, 3) appropriate use of medications, 4) communicating effectively with family, friends, and health professionals, 5) nutrition, and, 6) how to evaluate new treatments. It is the process in which the program is taught that makes it effective. Classes are highly participative, and mutual support builds the participants’ confidence in their ability to manage their health and maintain active and fulfilling lives. The CDSMP is the fastest growing EBPP in South Carolina. The Intervention Coordinator will also assist participants in the set menu control arm with enrollment in this program.

### Mail-delivered arthritis kit

The Arthritis Self-Management Tool Kit is packaged in a plastic envelope and contains 1) a “Self-Test” to help participants determine how arthritis affects their lives and self-tailor the use of the Tool Kit, including items related to pain, fatigue, physical limitations, and health worries; participants score this test themselves and are directed to specific parts of the Tool Kit based on their scores; 2) information sheets: Working with Your Doctor and the Health Care System; Exercise; Medications; Healthy Eating; Fatigue and Pain Management; Finding Community Resources; and Dealing with One’s Emotions; 3) information sheets on key process components of the ASMP: Action Planning, Problem Solving, Deciding What to Try, and Individualizing an Exercise Program; 4) *The Arthritis Helpbook* [81]; 5) audio relaxation and exercise compact discs (CDs); and 6) an audio CD of all material printed on the information sheets.

### Support group

Interested participants will be referred to the “Lupus: Listening and Learning Group”, a Charleston-based support group affiliated with the Lupus Foundation of America (LFA). One to two LFA-trained facilitators implement the group. Notices are placed in community papers, radio stations, and public access events calendars, as well as rheumatology offices and in emerging social networking modalities like Facebook. Additional outreach activities, such as presence at community health fairs, communication with local churches and networking with community-based organizations, would be welcomed in order to invite additional support group participants. The meeting format generally includes a specific discussion topic or an informative presentation such as by a medical or counseling professional, pharmacist, or lupus researcher. This program is followed by time within the group for further interaction and support among the attendees who wish to participate. The Lupus: Listening and Learning Group is open to all lupus patients, family members, friends and supporters, and there is no pre-registration or fee involved.

### Listserv

A project listserv will be established to link all intervention participants to facilitate exchange of coping strategies, pose questions, and share preferred educational resources or any other information relative to their everyday and disease-specific experiences and/or participation in the project. Participants will be provided with a URL, from which they will be able to subscribe to the listserv. Once subscribed, they will be able to post and receive messages, managed by a listserv moderator, who will be responsible for distribution to listserv members as appropriate. Participants will have the option to unsubscribe at any time.

### Handling of study interventions

To ensure intervention integrity, discrete components will follow written protocols and peer leaders will complete a standardized checklist for each session/encounter. Each IIP will be reviewed on a quarterly basis (every 3 months), at which time measures will be repeated and the participant will be invited to change and/or add options to their plan.

### Concomitant interventions

During the first intervention year, intervention arm participants will have the option of participating in a mail-delivered arthritis kit, addition and access to a listserv, participation in a support group, and/or enrollment in local self-management program(s). After one year, set menu controls and controls will be invited to participate in the same options that were originally extended to intervention participants. During the first intervention year, set menu control arm participants will not have the option of participating in a mail-delivered arthritis kit, addition and access to a listserv, or participation in a support group. Control arm participants will not have the option of participating in any of the four offered intervention modalities. None of the offered intervention modes are required.

### Adherence assessment

In addition to quarterly review of their IIP, intervention participants will receive biweekly follow up calls to gauge their progress, comfort, adherence, and occurrence of any adverse events. In addition to enrollment in a local CDSMP, participants in the set menu control arm will receive monthly follow up calls to gauge their progress, comfort, adherence, and occurrence of any adverse events. Set menu controls will complete post-intervention follow-up evaluations bi-annually. Participants in the control arm will not receive follow-up calls, but will complete post-intervention follow-up evaluations on the same schedule as set menu controls. At their second post-intervention follow-up evaluation (after one year), set menu controls and controls will be invited to participate in the intervention activities of the program and have the opportunity to create their own IIP with the same options that were originally extended to intervention participants. From then, all participants will review their plan with the intervention coordinator on a quarterly basis, at which time measures will be repeated and each participant will be invited to change and/or add options to their plan. All participants will then receive monthly follow up calls to gauge their progress, comfort, adherence, and occurrence of any adverse events.

### Outcomes assessments

Study assessments will be completed at baseline, 6 months, 12 months, 18 months, with a final evaluation at 24 months, for a total of five assessments. All patients who have agreed to participate will be mailed questionnaires for completion and compensation upon return, along with return postage and instructions for completing instruments. A randomly selected sub-sample (*n* = 50) of interested patients will be invited for assessment of urine catecholamine levels and urine collection instructions will be included in their mailed materials at baseline and 24 months/final evaluation. Mailed assessments will include: The Arthritis Self-Efficacy Scale, the Personal Health Questionnaire Depression Scale (PHQ-9), the Lupus Quality of Life Questionnaire (LUP-QOL), the Systemic Lupus Activity Questionnaire (SLAQ), and the Stanford Patient Education Research Center Questionnaires assessing medical outcomes such as hospital visits, illness intrusiveness, and use of stress management techniques.

### Primary outcome

We expect the proposed study to demonstrate that a unique intervention strategy can reduce disease activity and damage, as well as perceived and biological indicators of stress, while improving quality of life indicators in African-American lupus patients. This will result in changes (a) health behaviors (use of stress management techniques), (b) health status (quality of life, depression, perceived psychosocial stress, and disease activity), (c) health care utilization (hospitalizations, emergency room visits, and communication with physicians) and (d) biological markers (urinary catecholamines).

### Secondary outcome

Measures of psychosocial and neuroendocrine responses to stress and disease activity and damage indices are expected to be inversely related to participation in intervention activities. Indicators of quality of life are expected to be positively related to participation.

### Self-report measures

Standardized methods will be employed for measuring associated outcomes in a validated fashion. Multiple measures were chosen to assess the impact of the intervention on targeted outcomes and quality of life. The criteria for choosing instruments and techniques were that they have been previously validated (preferably in African American populations), represent key outcomes in one or more past studies of chronic conditions (preferably lupus), are relatively brief (no more than 50–60 questions total), and sensitive to change in the range of 0.2 effect size. Selected measurement instruments are described below. Depending on participant preference, questionnaires will be mailed (paper and electronic) or administered by telephone. All biological samples for analysis will be promptly processed in a Labcorp facility to ensure standardization of assessment.

### Psychosocial stress

Psychosocial stress will be assessed by the Arthritis Self-Efficacy Scale pain and other symptoms sub-scale [82], which consists of 11 items designed to measure confidence in one’s ability to manage the pain, fatigue, frustration, and other aspects of disease; it was reworded in previous investigations to reflect lupus rather than arthritis [39].

### Depression

Depression will be assessed by the PHQ-9 adapted by the Stanford Patient Education Research Center [83, 84]. The nine-item scale assesses the presence and frequency of depressive symptoms over the past two weeks, yielding a score range of 0–27 where a score of 15 or greater is considered major depression, and 20 or more is severe major depression.

### Quality of life

Quality of life will be assessed using two instruments that describe a spectrum of quality of life outcomes. The LUP-QOL incorporates the Medical Outcomes Study (MOS) Short Form 36 Health Survey (SF-36) and the Functional Assessment of Chronic Illness Therapy-Fatigue (FACIT-F), which are reliable and valid instruments that are frequently used in quality of life studies of persons with lupus [85–87]. The questionnaire includes questions pertaining to physical function, role function, social function, mental health, health perception and pain.

### Neuroendocrine response to stress

Assessment of urinary catecholamine levels will require that the identified sub-sample of participants collect their own samples in a brown 24-h urine container with 30 mL 6 N hydrogen chloride (HCl) of urine collected and the date/time collection started and finished. Samples will need to be kept refrigerated between collections and then the completed container kept on ice until couriers pick up samples and bring them back to the lab for testing. Random subjects will be asked for an immediate additional collection to assess variability imposed by participant-specific factors. A 4–30 mL aliquot is necessary for analysis. Assessment of urinary catecholamines includes tests of urinary epinephrine, norepinephrine, and dopamine, which will be quantified using liquid chromatography/tandem mass spectrometry (LC/MS-MS) [88–90]. Once results are complete, they will be sent via Autofax.

### Disease indices

Possible disease activity will be assessed using SLAQ [91]. The Systemic Lupus Activity Measure (SLAM) is a physician-rated, 31-item instrument that assesses symptoms and objective findings in the month prior to evaluation, in nine organ systems and seven laboratory items [92, 93]. Individual items are rated on a scale from 0 to 3 based on severity and are scored positively only if directly attributable to SLE. The SLAQ is based on items from the SLAM that could be self-reported. It asks a single Patient Global Assessment (PGA) question about presence and severity of lupus activity over the past month, questions on 24 specific symptoms of disease activity including weight loss, fatigue, fevers, oral ulcers, malar rash, photosensitivity, vasculitis, other rashes, alopecia, lymphadenopathy, dyspnea, chest pain, Raynaud’s phenomenon, abdominal pain, paresthesia, seizures, stroke, memory loss, depression, headaches, myalgias, muscle weakness, arthralgias, and joint swelling, and a single Numerical Rating Scale (NRS) asking the patient to rate disease activity on a scale of 0–10 over the past three months with the 0 anchored by ‘no activity’ and 10 anchored by ‘most activity’. The patient is then asked to rate the most active day over the past three months. Likert responses with four response categories (no problem, mild, moderate, severe) are used for the PGA and specific symptom questions.

To assess use of prednisone, immunosuppressive agents, nonsteroidal anti-inflammatory drugs [NSAIDs], and/or hydroxychloroquine), participants will also be asked to provide a listing of concomitant medications they are taking, to include the name of the medication, dose, unit, frequency, route, start date, stop date, and whether the medication will be ongoing at the end of the study. To account for factors that may affect catecholamine observations, participants will also be asked to include alcohol, caffeine, illegal/street drugs, and alternative remedies.

### Behavior change

Stanford Patient Education Research Center Questionnaires assessing medical outcomes such as hospital visits, illness intrusiveness, and use of stress management techniques [70, 77–79] will be used to further assess adoption and maintenance. These are behavior change scales, modified from the Medical Outcomes Study, to determine if participants are practicing cognitive stress reduction (pain reduction) and non-cognitive (mental stress management/ relaxation) techniques. These scales also assess whether key behaviors concerning communicating with health care providers and health care utilization have changed. Additionally, participants will have the opportunity to rate the credibility of the intervention and their expectancy of improvement. Participants will also be asked to keep diaries of practice during the intervention period. The number of diaries returned and self-ratings of practice frequency will be used to estimate adherence to intervention techniques and practices.

### Sample size and power

There will be three study arms in the proposed study; (1) intervention, (2) set menu control, and (3) control. Due to the potential similarity in each arm, a cluster-random trial will be considered, which takes into account between-cluster variation [94]. Relevant intervention literature shows effect sizes varying from 30-80 % [26, 29, 31, 39, 42, 45, 46, 49–51], which will be adequate for the proposed sample size design. The study will evaluate the difference in disease indices, quality of life and perceived and biological indicators of stress between the intervention and control groups, as well as within each group according to individual levels of intervention participation. Therefore, estimated sample sizes are displayed in Table 1 under a variety of effect sizes and drop-out levels in order to obtain a 0.95 significance level and 0.8 power of study. Our target recruitment level will be 150 participants according to an 80 % power level, in an effort to capture smaller effect sizes. This will correspond to an approximate 45 % response rate (*N* = 336), which is well within response and participation in other studies among the MUSC lupus cohort. Based on these results there is good to adequate power for most analyses to make the proposed project feasible.

**Table 1:** Estimated sample size calculations

Estimated sample sizes				
Effect size	0.4	0.6	0.7	0.8
Sample size per arm (No drop out)	51	23	18	14
Sample size per arm (10 % drop out)	86	38	28	22
Sample size per arm (15 % drop out)	91	41	30	23

### Data analytic plan

#### Quantitative data analysis

Data will be entered immediately after specified collection points into an Excel worksheet that will be built upon after each collection point and imported into SAS for data manipulation. To minimize risks to confidentiality, all data will be de-identified by assigning a numeric code to each participant and labeling all materials corresponding to that participant with their unique identifier. Additionally, data files that include personal identifiers will be stored separately from signed consent forms in locked file cabinets that only the Principal Investigator will have access to. The basic experimental design and corresponding data analyses will represent a one-between subjects (intervention versus control and intervention versus set menu control) and one-within or repeated factor (pre, post, and follow up assessments) crossed factorial design, with multiple dependent outcome measures (disease activity and damage, perceived and biological indicators of stress, quality of life, and urine catecholamines). The multivariate (multiple qualitatively distinct measures at multiple times) approaches to repeated measures [95, 96], based on the general linear model or general linear mixed model, will be used to test for pre-post-follow up changes between the intervention group and the control groups, as well as for exploratory correlations with urine catecholamines. These approaches provide better description of longitudinal patterns for each dependent variable and they are easier to successfully implement and generally more powerful than the use of covariant models when multiple outcome measures are considered simultaneously [96]. *P* < 0.05 will be used to indicate statistical significance. The data from all randomized participants will be analyzed. Multiple imputations will be used to estimate missing data from dropouts and those participants who do not complete follow up evaluations. We will evaluate the first 12 months to address primary outcomes and exploratory analyses will be conducted after 12 months once participants are given the option to cross over. Basic descriptive statistics such as means, ranges, and standard deviations will be calculated and tabulated for quantitative data using SAS (SAS Institute, Cary, North Carolina). Project-specific frequencies and percentages will be presented as a recruitment diagram according to CONSORT guidelines.

### Qualitative data analysis

Qualitative data will be analyzed using QSR NVivo 8 (Qualitative Solutions and Research, Pty Ltd, Victoria, Australia), a software program for managing, coding, analyzing, and retrieving qualitative data. Data will be coded electronically in NVivo 8 and printed out by code, reviewed for accuracy, and examined for links to other codes. This “axial coding” [95] process will connect code categories and identify relationships between codes which are suggestive of themes. Furthermore, as part of the ongoing analytic process, comparing and contrasting themes within and across the two strata (“constant comparison method”) will detect similarities and differences in the data [95]. Verbatim quotes will be selected to validate researchers’ coding and interpretations. The Z test and Student’s *t* test will be used for continuous variables satisfying normality assumption, and Mann Whitney *U* test and Kruskal-Wallis Test will be applied for variables which cannot satisfy the normal assumption. All statistical comparison tests will be at a 95 % significance level.

### Safety monitoring

Study procedures and participants will be monitored throughout the course of the project to ensure patient safety and minimal risk. Intervention and study coordinators will monitor study participants at consent, all data collection encounters, and during intervention activities for any discomfort, satisfaction with study participation, and for any questions or concerns participants may have. At critical data collection junctures, the PI will review the data set to ensure de-identification prior to analysis. On a daily basis, the PI and Biostatistician will ensure the security of all data files. The National Institute of Arthritis And Musculoskeletal And Skin Diseases (NIAMS)-appointed Safety Officer (SO), Dr. Belmont, along with the Data and Safety Monitoring Committee (DSMC), which will consist of Doctors Gary Gilkeson, Holly Mitchell, and Diane Kamen, will review safety and study conduct information on a semi-annual basis or on an ad hoc basis should safety or privacy concerns be raised. NIAMS determines the frequency of safety reports that are submitted to the Safety Officer. The frequency is most often bi-annually. The report is submitted to KAI Research, Inc. (KAI) and KAI will then inform NIAMS and the SO that it is available for their review.

Any adverse events (AE) or serious adverse events (SAE) will be communicated to the USC IRB. AEs will be collected during each follow-up phone call by prompting participants to let the Intervention Coordinator and/or GA know if they have experienced any AE. In addition, should events be reported in-between follow-up calls, those will be recorded immediately. The PI will be notified of all AEs and SAEs. All SAEs will be reported to the independent SO through KAI and NIAMS within 48 h of the PI becoming aware of the event.

The study will only be stopped in the event of an unfavorable safety review. Safety findings that might trigger a safety review and would temporarily suspend enrollment and/or study intervention until a safety review is convened include the number of SAEs overall, the number of occurrences of a particular type of SAE, severe AEs/reactions, or increased frequency of events. Such findings will be presented to the study biostatistician or to the DSMC to review the events by group to determine whether there are statistical as well as clinical concerns. The statistician reports his findings to a closed session of the DSMC or to the SO and/or NIA. The findings are used to determine whether the study should continue per protocol, proceed with caution, be further investigated, be discontinued, or be modified and then proceed.

Participants will always be given the option of discontinuing participation in the event they are unhappy with further participation. This is at the discretion of the participant. Any participant assigned to the intervention or set menu study arms during Year 1 who fails to complete 3 or more of the 6 CDSMP/ASMP sessions and/or fails to select and regularly participate in at least one other intervention choice (listserv, support group, mailed kit) for at least three consecutive months, will be removed from their respective intervention arm and coded/analyzed as a control participant since non-participation will render them closer to the control or usual standard of care arm. The study may be discontinued at any time by the IRB, the NIA, the OHRP, the FDA, or other government agencies as part of their duties to ensure that research participants are protected.

A steering committee of seven mentors (JCO, KL, SHG, DK, SB, SW, and ATM) will provide intensive mentorship to the PI (EMW) and will be responsible for the continued integrity of the intervention program and its representation of USC and all affiliate organizations, programs and institutions. Publication of the results of this trial will be governed by the policies and procedures developed by the Steering Committee. Any presentation, abstract, or manuscript will be made available for review by the sponsor and NIAMS prior to submission.

## Discussion

Results from this trial will provide important insight into the design and effectiveness of a culturally sensitive educational intervention which includes self-selection of program components aiming to improve quality of life and health status among African American patients with lupus. We have built on three decades of empirical work conducted in the field of arthritis self-management, but differs in that the intervention mode, the disease (lupus), and the study population (African Americans) are unstudied or understudied. This psycho/educational intervention is unique in that it will be a la carte. It will allow each participant to choose which mode or modes by which they care to participate. These modes include small group, mail, and/or listserv discussion group. Such a culturally sensitive educational intervention which includes self-selection of program components has the potential to improve disparate trends in quality of life, disease activity and depression, and stress among African American lupus patients, as better self-management outcomes have been documented when participants are able to choose/dictate the content and/or pace of the respective treatment/intervention program [68–71]. Since there is currently no “gold standard” self-management program specifically for SLE, the IQAN project may have a considerable impact on future research and policy decisions. Since this proposal addresses a health disparities need in the field of lupus, wide-spread implementation in urban communities that have large populations of underserved minorities who would benefit from such an interventions is an attainable goal.

### Trial status

Currently, 153 participants have been randomized and 30 have completed all assessments.

## Abbreviations

ACR, American College of Rheumatology; AFSHP, Arthritis Foundation Self-Help Program; ASMP, arthritis self-management program; CBSM, cognitive-behavioral stress management; CD, compact disc; CDSMP, Chronic Disease Self-Management Program; CNS, central nervous system; DNA, deoxyribonucleic acid; DSMC, Data and Safety Monitoring Committee; EBPP, Evidence based prevention program; FACIT-F, functional assessment of chronic illness therapy-fatigue; FDA, Food and Drug Administration; GA, graduate assistant; HCl, hydrogen chloride; IIP, individualized intervention plan; IQAN, intervention to improve quality of life for african-american lupus patients; IRB, Institutional Review Board; KAI, KAI Research, Inc.; LC/MS-MS, liquid chromatography/tandem mass spectrometry; LFA, Lupus Foundation of America; LUP-QOL, lupus quality of life questionnaire; MOS, medical outcomes study; MUSC, Medical University of South Carolina; NIA, National Institute on Aging; NIAMS, National Institute of Arthritis and Musculoskeletal and Skin Diseases; NRS, numerical rating scale; NSAIDS, Non-steroidal anti-inflammatory drugs; OHRP, Office for Human Research Protections; PGA, patient global assessment; PHQ-9, personal health questionnaire depression scale; PI, Principal Investigator; RE-AIM, reach, effectiveness, adoption, implementation, and maintenance; RNA, ribonucleic acid; SCDHEC, South Carolina Department of Health and Environmental Control; SCLGOA, South Carolina Lieutenant Governor's Office Administration; SF-36, short form 36 health survey; SLAM, systemic lupus activity measure; SLAQ, systemic lupus activity questionnaire; SLE, systemic lupus erythematosus; SLEIGH, SLE in Gullah health; SO, safety officer; UC, usual care; USC, University of South Carolina
